# Bridging the Gap: Exploring the Synergy Between Pet Economy and Super-Aged Societies for Sustainable Well-being

**DOI:** 10.1093/geroni/igaf122.2328

**Published:** 2025-12-31

**Authors:** Chen-Chang Su

**Affiliations:** National Chung Cheng University., Minxiong, Chiayi, Taiwan (Republic of China)

## Abstract

As global populations rapidly age, the social and economic challenges of super-aged societies—such as loneliness, rising healthcare costs, and fragmented community ties—demand innovative solutions. Simultaneously, the pet economy, driven by companion animals and pet-related technologies, has emerged as a potential catalyst for addressing these challenges. This study investigates the dual role of pets as emotional companions and economic drivers in aging populations, employing a mixed-methods approach combining quantitative analysis of national health surveys (N = 5,000 elderly participants) and qualitative interviews with stakeholders (pet owners, industry leaders, and policymakers). Preliminary findings indicate that pet ownership is significantly associated with reduced loneliness scores (β = −0.32, p < 0.01) and lower annual healthcare expenditures (15% decrease compared to non-owners). Furthermore, case studies from Japan and Germany highlight successful models of pet-inclusive senior housing and robotic pet adoption programs. The research proposes a framework for “pet-integrated aging ecosystems,” emphasizing cross-sector collaboration among healthcare, technology, and urban planning. This study contributes to gerontology and consumer behavior literature by bridging the gap between silver economy strategies and pet industry innovations, while offering actionable insights for policymakers aiming to leverage pet-related interventions in aging societies.

